# Comparison of cardiovascular disease and cancer prevalence between Mediterranean and north European middle-aged populations (The Cilento on Ageing Outcomes Study and The Malmö Offspring Study)

**DOI:** 10.1007/s11739-020-02625-4

**Published:** 2021-01-30

**Authors:** Olle Melander, Paola Antonini, Filip Ottosson, Louise Brunkwall, Widet Gallo, Peter M. Nilsson, Marju Orho-Melander, Gaetano Pacente, Giovanni D’Arena, Salvatore Di Somma

**Affiliations:** 1 Department of Clinical Sciences Malmö, Lund University, CRC, Jan Waldenströms gata 35, 21428 Malmö, Sweden; 2 Department of Emergency and Internal Medicine, Skåne University Hospital, Malmö, Sweden; 3 Department of Medical-Surgery Sciences and Translational Medicine, University of Rome Sapienza, Rome, Italy; 4 GREAT Health Sciences, Rome, Italy; 5 D’Arena Laboratory, Vallo della Lucania, Salerno, Italy

**Keywords:** Cardiovascular disease, Cancer, Mediterranean diet, Cardiometabolic risk factors

## Abstract

Mediterranean diet protects from both cardiovascular disease (CVD) and cancer. In the 1960s, Ancel Keys defined the concept of Mediterranean diet in the South Italian region of Cilento and proposed it as a key factor for healthy ageing in the region. The aim of the current study was to compare the prevalence of CVD and cancer between a middle-aged population from Cilento and those of a Northern European population from Malmö, Sweden. We clinically characterized two middle-aged (50–67 years of age) population-based samples from Cilento (*n* = 809) and Malmö (*n* = 1025), Sweden, respectively. Logistic regression was used to calculate odds ratios (95% confidence interval) for disease prevalence in Malmö versus Cilento inhabitants adjusted for age and sex (model 1) and adjusted for all cardiometabolic risk factors (model 2). The prevalence of hypertension, current smoking, diabetes mellitus and levels of body mass index and triglycerides were lower, whereas HDL-cholesterol was higher in Malmö than in Cilento. LDL-cholesterol was higher and estimated glomerular filtration rate was lower in Malmö than in Cilento. The odds ratio for cardiovascular disease in Malmö versus Cilento inhabitants was 1.13 (0.69–1.87) (*P* = 0.62) in model 1, whereas it was significantly elevated in model 2 [2.03 (1.14–3.60) (P = 0.016)]. Moreover, the odds ratio for cancer in Malmö versus Cilento was 2.78 (1.81–4.27) (*P* < 0.001) in model 1 and 3.11 (1.97–4.92) (*P* < 0.001) in model 2. The higher odds of CVD and cancer in Malmö versus Cilento, when risk factors were accounted for, suggests the existence of unknown protective factors in Cilento.

## Background

Cardiovascular disease (CVD) and cancer are the most common non-communicable diseases globally and account for the majority of morbidity, disability, impaired quality of life and reduction of life span [1, 2]. In addition to non-modifiable risk factors such as age and sex, the strongest risk factors/markers are related to unfavorable lifestyle elements such as cigarette smoking, psychosocial factors, low intake of fruits and vegetables and physical inactivity [3]. CVD mortality has fallen over the past 10-year period, which has been attributed to, among other things, a reduction of cigarette smoking, more effective prevention of high blood pressure and cholesterol and new treatment methods for myocardial infarction and stroke [1]. However, both in Europe and globally there is a clear increase in obesity and type 2 diabetes, a trend that threatens our future health as obesity and diabetes are risk factors for both CVD and cancer [2, 4–7].

There is a well-known gradient of cardiovascular mortality across Europe, with lowest rates in the southwest [8, 9]. Moreover, individuals who follow a Mediterranean diet, which includes a large proportion of fruits and vegetables, olive oil, fish and a moderate daily intake of wine, have a lower risk of both CVD and cancer [2, 10–12]. The exact causes of this protective effect are still unknown but are probably related not only to the diet itself but also to other co-varying lifestyle factors.

Cilento, a rural area of the Campania region in South Italy, about 130 km south of Naples, has a typical Mediterranean climate, and this is where the American cardiovascular scientist Ancel Keys first described the Mediterranean Diet and proposed it as a key factor contributing to healthy ageing in the region [13]. Recently, it was shown that nonagenarians and centenarians (NCs) from Cilento had better mental well-being than their younger family members [14]. Moreover, NCs had lower low-density lipoprotein cholesterol (LDL-C) and fasting glucose levels compared to middle-aged family members.[15]. As the study of very old people might be subject to survival bias, the current study was focused on disease prevalence and risk factors in middle-aged populations.

The aim of this study was to compare the prevalence of cardiometabolic risk factors, CVD and cancer between a large middle-aged, random sample of the Cilento population with a similar middle-aged population-based sample from Malmö, Sweden. To obtain comparable populations, the protocols used in the two studies were synchronized with, e.g. similar selection tools, study visits, exams and identical questionnaires (except for language).

## Methods

### Study populations

A random sample of Cilento middle-aged (50–67 years) individuals was obtained by invitations based on all individuals listed at local primary health care centres in 24 representative Cilento villages. All Cilento inhabitants are listed at a primary health care centre, regardless of whether any healthy issue ever occurred or not. The order of lists including all subjects at each of the primary health care centres within the defined age span were randomized. Subsequently, subjects were invited (in randomized order) by letter and telephone to ask for willingness to participate. The participation rate was 55% resulting in the inclusion of 1008 individuals in the given age span.

In Malmö, a corresponding population sample (the Malmö Offspring Study)[16] was acquired by invitations by letter and telephone to individuals who were adult offspring of participants in a prior population-based health survey study of the city of Malmö called the Malmö Diet and Cancer Study [17]. From the Malmö Offspring Study, we included the subjects within the same age range as in Cilento, i.e. 50–67 years. This resulted in 1184 included individuals (participation rate 50%) in the age span in question.

### Clinical characterization

We applied identical questionnaires in both populations apart from language, i.e. the questionnaire used in the Malmö Offspring Study was translated into Italian by a professional translator and used for Cilento study participants. The design of the Malmö Offspring Study was recently published [18] and its questionnaire is derived from the validated questionnaire used in the Malmö Diet and Cancer Study [19]. Data from the questionnaire, which include common diseases, occupation status, physical activity, smoking, alcohol, food frequency and sleep, were used to obtain data on smoking habits (never smoked, stopped smoking, current smoking) and prevalence of cancer, CVD (myocardial infarction or stroke), hypertension, diabetes and dyslipidemia. Prevalence of these diseases/conditions was defined as answering yes on the question of whether they had ever been physician diagnosed and/or treated for the disease/condition in question. The sensitivity of self-reported myocardial infarction and stroke, when compared to hospital discharge diagnoses, has been shown to be > 90% and > 80%, respectively [20].

Moreover, at both sites, a clinical examination, following the same work-flow and methods as described below, was performed. Participants came to the respective clinic (one centralized clinic in Cilento and one in Malmö) after an overnight fast since 22:00 the evening before. Height, weight and waist circumference were recorded in light indoor clothing, and after 5 min rest, blood pressure was measured in the seated position. Fasting plasma glucose was measured using the glucose dehydrogenase method with photometric using a Hemocue Glucose 201 + device (Hemocue, Ängelholm, Sweden). Levels of LDL-C and HDL-C, as well as triglycerides and creatinine, were measured on automated and accredited devices at the respective sites. Estimated glomerular filtration (eGFR) rate was calculated according to the modification of diet in renal disease study equation (MDRD) formula [21]. In addition, a biobank of EDTA plasma and stool samples was stored for future studies in −80 degree freezers. All data were collected and stored into a RedCap database v9.1.0, Vanderbilt University, TN.

### Statistics

Continuous variables are expressed as means ± standard deviation and were compared between the two populations with univariate statistics using independent samples *t* test, and in age- and sex-adjusted analyses, linear regression was used. For comparison of categorical variables between the two populations, chi-squared test was used in univariate statistics and logistic regression was used for corresponding multivariate models with disease prevalence (no = 0, yes = 1) as the dependent variable and living in Cilento or Malmö (Cilento = 0, Malmö = 1) (key exposure) along with other covariates, as specified in the results, as independent variables. A *P* value less than 0.05 was considered significant. SPSS statistical software version 26 (IBM, Chicago, IL) was used for all calculations.

### Ethics

All subjects provided written informed consent. The Malmö Offspring Study was approved by the Regional Board of Ethics in Lund (Dnr 2012/594) and the Cilento on Ageing Outcomes Study was approved by the Regional Board of Ethics ASL Napoli Sud (20171220).

## Results

### Demographics and risk factors

Of the 2192 study participants, we excluded subjects who had missing values either due to incomplete questionnaire or missing value on any of the clinical measurements (*n* = 358), resulting in a study population of 1834 subjects with complete data on all variables. Clinical characteristics are shown in Table 1. There was no difference in age or gender distribution between the Malmö and Cilento cohorts. Hypertension and diabetes mellitus were significantly less common in Malmö than in Cilento, both in crude and in age- and sex-adjusted models. In line with these findings, systolic blood pressure (but not diastolic blood pressure), body mass index, fasting plasma glucose and triglycerides were lower, but HDL-cholesterol higher, in Malmö than in Cilento. Whereas current smoking was approximately twice as high in Cilento as in Malmö, the rate of subjects who had smoked previously but stopped was higher in Malmö than in Cilento (38.8% vs 26.7%). As a result of this, the rate of ever smokers did not differ between the two cohorts. Finally, the Malmö as compared to the Cilento population had significantly lower eGFR and higher LDL-cholesterol (Table 1).

**Table 1:** Clinical characteristics of Mediterranean (Cilento, Italy) and North European (Malmö, Sweden) middle-aged study populations

	Cilento (*n* = 809)	Malmö (*n* = 1025)	*P* value (crude)	*P* value (adjusted)*
Age (years)	57.7 ± 4.6	57.4 ± 4.1	NS	NS
Men, *n* (%)	369 (45.6)	484 (47.2)	NS	NS
BMI (kg/m^2^)	28.3 ± 5.4	27.2 ± 4.8	< 0.001	< 0.001
Obesity, *n* (%)	240 (29.7)	263 (25.7)	0.056	0.054
SBP (mmHg)	131 ± 15	129 ± 17	0.003	0.004
DBP (mmHg)	77.8 ± 9.3	80.0 ± 9.8	< 0.001	< 0.001
Hypertension, *n* (%)	352 (43.5)	384 (37.5)	0.009	0.017
Glucose (mmol/L)	5.9 ± 2.0	5.7 ± 1.2	0.005	0.005
Diabetes, *n* (%)	75 (9.3)	59 (5.8)	0.004	0.007
HDL-C (mmol/L)	1.53 ± 0.40	1.71 ± 0.55	< 0.001	< 0.001
LDL-C (mmol/L)	3.23 ± 0.82	3.54 ± 0.93	< 0.001	< 0.001
LDL-C > 2.6 mmol/L, *n* (%)	634 (78.4)	891 (86.9)	< 0.001	< 0.001
TG (mmol/L)	1.43 ± 0.91	1.28 ± 0.77	< 0.001	< 0.001
TG > 1.7 mmol/L, *n* (%)	207 (25.6)	203 (19.8)	0.003	0.002
eGFR (mL/min)	86.6 ± 18.0	83.7 ± 15.9	< 0.001	< 0.001
Current smoker, *n* (%)	209 (25.8)	132 (12.9)	< 0.001	< 0.001
Ever smoker, *n* (%)	425 (52.5)	530 (51.7)	NS	NS

*BMI* body mass index, *SBP* systolic blood pressure, *DBP* diastolic blood pressure, *HDL-C* high-density lipoprotein cholesterol, *LDL-C* low-density lipoprotein cholesterol, *TG* triglycerides, *eGFR* estimated glomerular filtration rate

^*^Adjusted for age and sex

### Cardiovascular disease and cancer prevalence in Malmö and Cilento

**Fig. 1:**
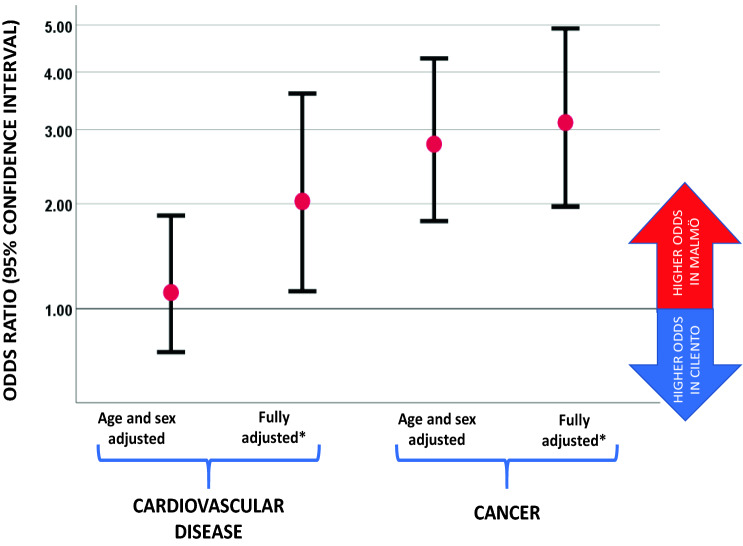
Odds ratios (95% confidence intervals) for cardiovascular disease and cancer in Malmö (odd ratios above 1) compared with Cilento (odds ratios below 1). *Fully adjusted model includes age, sex, body mass index, systolic and diastolic blood pressure, hypertension, diabetes, glucose, smoking habits, eGFR, LDL-C, HDL-C and triglycerides

In Malmö, 39 subjects (3.8%) had been diagnosed with CVD (*n* = 22 with myocardial infarction and *n* = 20 with stroke, of whom *n* = 3 had had both myocardial infarction and stroke) and in Cilento 28 subjects reported CVD (*n* = 19 with myocardial infarction and *n* = 10 with stroke, of whom *n* = 1 had had both myocardial infarction and stroke). As shown in Fig. 1, no significant difference was found for the age- and sex-adjusted odds ratio (95% confidence interval) for prevalent CVD in Malmö versus Cilento that was 1.13 (0.69–1.87) (*P* = 0.62). When adjusted for the differences in risk factor levels between the two populations on top of age and sex (body mass index, systolic and diastolic blood pressure, hypertension, diabetes, glucose, smoking habits, eGFR, LDL-C, HDL-C and triglycerides), the odds for cardiovascular disease was significantly higher in Malmö versus Cilento with an odds ratio (95% confidence interval) of 2.03 (1.14–3.60) (*P* = 0.016) (Fig. 1). Within the multivariate-adjusted model, the strongest factors associated with higher CVD prevalence were hypertension [2.32 (1.36–4.26) (*P* = 0.007)] and current smoking [2.16 (1.04–4.50) (*P* = 0.039)]. When substituting continuous values of risk factors with their categorized counterparts in the multivariate-adjusted model, the odds ratio for CVD (95% confidence interval) in Malmö versus Cilento was 1.88 (1.06–3.32) (*P* = 0.030).

Moreover, in Malmö, the number of subjects previously diagnosed with cancer was 33 (6.8%) as compared with 13 (1.5%) in Cilento. As shown in Fig. 1, the age- and sex-adjusted odds ratio (95% confidence interval) for cancer prevalence in Malmö versus Cilento was 2.78 (1.81–4.27) (*P* < 0.001). This higher odds for cancer prevalence in Malmö versus Cilento remained after further adjustment for age, sex and cardiometabolic risk factors (age, sex, body mass index, systolic and diastolic blood pressure, hypertension, diabetes, glucose, smoking habits, eGFR, LDL-C, HDL-C and triglycerides) with odds ratio of 3.11 (1.97–4.92) (*P* < 0.001). After stratification for sex, the corresponding odds ratios for cancer in Malmö versus Cilento were 2.34 (1.12–4.89) (*P* = 0.023) in men and 3.70 (2.02–6.81) (*P* < 0.001) in women (Fig. 1).

## Discussion

The key finding of this study is that in comparison with Malmö, a Northern European middle-aged population, the middle-aged population from the Mediterranean region of Cilento, have similar prevalence rates of CVD despite the presence of more risk factors, and when risk factors are accounted for, the odds for CVD is about half in Cilento as compared to Malmö. Moreover, the prevalence of cancer in Cilento was about one-third as compared to Malmö, both before and after accounting for risk factors. Our study is the first in its kind to compare CVD and cancer prevalence between a typical Mediterranean diet area and a Northern European city, using identical questionnaires and synchronized protocols.

Whereas current smoking was approximately twice as common in Cilento as in Malmö, the proportion of individuals who had ever smoked in Cilento was similar to that in Malmö due to the larger number of individuals who had quit smoking in Malmö. Moreover, hypertension and diabetes were more common in Cilento, along with higher body mass index, triglycerides and lower HDL-C. On the other hand, the Cilento population had lower LDL-cholesterol and better renal function as assessed by eGFR. In the multivariate analyses, hypertension and smoking were the strongest risk factors for CVD. Given the similar prevalence of CVD, and the higher odds for CVD in Malmö when risk factors were accounted for, we suggest that the Cilento population might be protected from CVD by non-measured factors, which are over-ruled by more current smokers and more hypertension. Of note, our study is cross-sectional and non-interventional and cannot address the role of Mediterranean diet itself in the difference of disease prevalence and risk factors between the two populations. However, it encourages more studies aimed at a deeper search for novel lifestyle-related risk and protective factors in the two populations.

We identified a marked difference in cancer prevalence, with about three times higher prevalence in Malmö as compared to Cilento in both age- and sex-adjusted and in fully adjusted models. Given the fact that the two strongest risk factors for cancer, apart from age, i.e. higher body mass index and smoking were more prominent in Cilento, this finding is highly surprising. It further emphasizes the possibility of unknown “hidden” protective factors in Cilento and encourages further studies aimed at the identification of lifestyle-related and biological factors in Cilento and their role in protection from cancer.

Our study has several limitations. The number of CVD and cancer cases was limited, leading to low robustness of the analyses. Data on disease prevalence were based on self-report which is a clear limitation, given imperfect validity. The sensitivity of self-reported myocardial infarction and stroke, when compared to hospital discharge diagnoses, is approximately 90% and 80%, respectively [20]. Total cholesterol values are missing which constitutes a limitation; however, we did adjust models for triglycerides, LDL- and HDL-cholesterol. Most importantly, the study is observational and cross-sectional and thus we cannot make any conclusions on causality. Moreover, the main outcomes of CVD and cancer prevalence might be affected by survival bias. For survival bias to explain our result of a threefold higher prevalence of cancer in Cilento than in Malmö, a substantially greater mortality in cancer at early ages in Cilento than in Malmö would be required. We believe this is very unlikely since only a minority of deaths due to cancer in general occur before the age of 50 years [22].

Another potential source of bias contributing to the lower prevalence of cancer and the lower odds of CVD in multivariate models in Cilento would be a more pronounced healthy cohort effect in Cilento than in Malmö, which might lead to problems of representativity. We find this unlikely given the similar sampling strategies resulting in balanced age and gender distribution in the two cohorts. Moreover, the higher rates of smoking, hypertension and diabetes in Cilento also strongly argue against a healthy cohort bias in Cilento exceeding that of the Malmö cohort.

An additional source of bias that could contribute to the markedly higher prevalence of cancer in Cilento than in Malmö would be more active and frequent screening for cancer in Malmö than in Cilento. National screening for breast and cervical cancer in women is long since routine in both Italy and Sweden, whereas none of the two countries had national programs for prostate cancer screening at that time [23–26]. This strongly argues against differences in cancer screening routines as an explanation for the threefold higher prevalence of cancer in Malmö as compared to Cilento. The validity of the finding is also supported by its presence in both males and females.

We conclude that the middle-aged population of the Mediterranean region of Cilento has markedly lower prevalence of cancer, similar CVD prevalence despite more traditional risk factors and lower odds for CVD when risk factors are accounted for, when compared with a Northern European population of Malmö examined with similar study protocols. Our findings point at the existence of unidentified protective factors in Cilento, encouraging further studies aimed at the identification of such factors.

## References

[1] Townsend N, Wilson L, Bhatnagar P, Wickramasinghe K, Rayner M, Nichols M (2016). Cardiovascular disease in Europe: epidemiological update 2016. Eur Heart J.

[2] WHO (2014). Global status report on noncommunicable disease 2014.

[3] Yusuf S, Hawken S, Ounpuu S, Dans T, Avezum A, Lanas F, McQueen M, Budaj A, Pais P, Varigos J, Lisheng L, Investigators IS (2004). Effect of potentially modifiable risk factors associated with myocardial infarction in 52 countries (the INTERHEART study): case-control study. Lancet.

[4] Colditz GA, Peterson LL (2018). Obesity and cancer: evidence, impact, and future directions. Clin Chem.

[5] Coutinho M, Gerstein HC, Wang Y, Yusuf S (1999). The relationship between glucose and incident cardiovascular events. A metaregression analysis of published data from 20 studies of 95,783 individuals followed for 12.4 years. Diabetes Care.

[6] Laakso M (1999). Hyperglycemia and cardiovascular disease in type 2 diabetes. Diabetes.

[7] Zimmet P, Alberti KG, Shaw J (2001). Global and societal implications of the diabetes epidemic. Nature.

[8] Sans S, Kesteloot H, Kromhout D (1997). The burden of cardiovascular diseases mortality in Europe. Task Force of the European Society of Cardiology on cardiovascular mortality and morbidity statistics in Europe. Eur Heart J..

[9] Muller-Nordhorn J, Binting S, Roll S, Willich SN (2008). An update on regional variation in cardiovascular mortality within Europe. Eur Heart J.

[10] Estruch R, Ros E, Salas-Salvado J, Covas MI, Corella D, Aros F, Gomez-Gracia E, Ruiz-Gutierrez V, Fiol M, Lapetra J, Lamuela-Raventos RM, Serra-Majem L, Pinto X, Basora J, Munoz MA, Sorli JV, Martinez JA, Fito M, Gea A, Hernan MA, Martinez-Gonzalez MA, Investigators PS (2018). Primary prevention of cardiovascular disease with a Mediterranean diet supplemented with extra-virgin olive oil or nuts. N Eng J Med.

[11] Tong TY, Wareham NJ, Khaw KT, Imamura F, Forouhi NG (2016). Prospective association of the Mediterranean diet with cardiovascular disease incidence and mortality and its population impact in a non-Mediterranean population: the EPIC-Norfolk study. BMC Med.

[12] Freisling H, Viallon V, Lennon H, Bagnardi V, Ricci C, Butterworth AS, Sweeting M, Muller D, Romieu I, Bazelle P, Kvaskoff M, Arveux P, Severi G, Bamia C, Kuhn T, Kaaks R, Bergmann M, Boeing H, Tjonneland A, Olsen A, Overvad K, Dahm CC, Menendez V, Agudo A, Sanchez MJ, Amiano P, Santiuste C, Gurrea AB, Tong TYN, Schmidt JA, Tzoulaki I, Tsilidis KK, Ward H, Palli D, Agnoli C, Tumino R, Ricceri F, Panico S, Picavet HSJ, Bakker M, Monninkhof E, Nilsson P, Manjer J, Rolandsson O, Thysell E, Weiderpass E, Jenab M, Riboli E, Vineis P, Danesh J, Wareham NJ, Gunter MJ, Ferrari P (2020). Lifestyle factors and risk of multimorbidity of cancer and cardiometabolic diseases: a multinational cohort study. BMC Med.

[13] Keys A and M. K. Eat well and stay well. Doubleday; 1st American Edition edition (January 1963). 1963.

[14] Scelzo A, Di Somma S, Antonini P, Montross LP, Schork N, Brenner D, Jeste DV (2018). Mixed-methods quantitative-qualitative study of 29 nonagenarians and centenarians in rural Southern Italy: focus on positive psychological traits. Int Psychogeriatr.

[15] Daniels LB, Antonini P, Marino R, Rizzo M, Navarin S, Lucibello SG, Maisel AS, Pizza V, Brenner DA, Jeste DV, Di Somma S (2020). Cardiovascular health of nonagenarians in southern Italy: a cross-sectional, home-based pilot study of longevity. J Cardiovasc Med.

[16] Ottosson F, Brunkwall L, Ericson U, Nilsson PM, Almgren P, Fernandez C, Melander O, Orho-Melander M (2018). Connection between BMI-related plasma metabolite profile and gut microbiota. J Clin Endocrinol Metab.

[17] Melander O, Newton-Cheh C, Almgren P, Hedblad B, Berglund G, Engstrom G, Persson M, Smith JG, Magnusson M, Christensson A, Struck J, Morgenthaler NG, Bergmann A, Pencina MJ, Wang TJ (2009). Novel and conventional biomarkers for prediction of incident cardiovascular events in the community. JAMA.

[18] Brunkwall L, Jonsson D, Ericson U, Hellstrand S, Kennback C, Ostling G, Jujic A, Melander O, Engstrom G, Nilsson J, Ohlsson B, Klinge B, Orho-Melander M, Persson M, Nilsson PM (2020). The Malmo Offspring Study (MOS): design, methods and first results. Eur J Epidemiol.

[19] Berglund PG (1993). Minisymposium: The Malmo Diet and Cancer Study Design, biological bank and biomarker programme. 23 October 1991 Malmo, Sweden. J Intern Med.

[20] Eliassen BM, Melhus M, Tell GS, Borch KB, Braaten T, Broderstad AR, Graff-Iversen S (2016). Validity of self-reported myocardial infarction and stroke in regions with Sami and Norwegian populations: the SAMINOR 1 survey and the CVDNOR project. BMJ Open.

[21] Levey AS, Coresh J, Greene T, Stevens LA, Zhang YL, Hendriksen S, Kusek JW, Van Lente F, Chronic Kidney Disease Epidemiology C (2006). Using standardized serum creatinine values in the modification of diet in renal disease study equation for estimating glomerular filtration rate. Ann Intern Med..

[22] UK CR. Cancer mortality by age. https://www.cancerresearchukorg/health-professional/cancer-statistics/mortality/age#ref. 2019.

[23] Puliti D, Bucchi L, Mancini S, Paci E, Baracco S, Campari C, Canuti D, Cirilli C, Collina N, Conti GM, Di Felice E, Falcini F, Michiara M, Negri R, Ravaioli A, Sassoli De’ Bianchi P, Serafini M, Zorzi M, Caldarella A, Cataliotti L, Zappa M, Group ICW (2017). Advanced breast cancer rates in the epoch of service screening: the 400,000 women cohort study from Italy. Eur J Cancer..

[24] Hortlund M, Elfstrom KM, Sparen P, Almstedt P, Strander B, Dillner J (2018). Cervical cancer screening in Sweden 2014–2016. PLoS ONE.

[25] Haukka J, Byrnes G, Boniol M, Autier P (2011). Trends in breast cancer mortality in Sweden before and after implementation of mammography screening. PLoS ONE.

[26] Cappelli MG, Fortunato F, Tafuri S, Boccalini S, Bonanni P, Prato R, Martinelli D (2018). Cervical cancer prevention: an Italian scenario between organised screening and human papillomaviruses vaccination. Eur J Cancer Care.

